# Factors associated with carotid Intima media thickness and carotid plaque score in community-dwelling and non-diabetic individuals

**DOI:** 10.1186/s12872-018-0752-1

**Published:** 2018-02-06

**Authors:** Javad Alizargar, Chyi-Huey Bai

**Affiliations:** 1 School of Public Health, College of Public Health, Taipei Medical University, 250 Wu-Hsing Street, Taipei City, 11031 Taiwan; 2 Department of Public Health, College of Medicine, Taipei Medical University, 250 Wu-Hsing Street, Taipei City, 11031 Taiwan

**Keywords:** Carotid Intima media thickness, Atherosclerosis, Hemoglobin A, Glycosylated, Blood urea nitrogen

## Abstract

**Background:**

The carotid intima media thickness (cIMT) and carotid plaque score (cPS) are respective markers of early and late stage subclinical atherosclerosis. Relationships between some laboratory parameters and subclinical atherosclerosis are not yet clear in community dwelling individuals and non-diabetic subjects, so we try to elucidate these relationships and find a model to predict early and late stage subclinical atherosclerosis.

**Methods:**

We examined relationships of the cIMT and cPS with different laboratory and demographic data of 331 subjects from a community-based prospective cohort study, using univariate and multivariate analyses.

**Results:**

In regression models and after multiple adjustments, only systolic blood pressure (SBP), age, glycated hemoglobin (HBA1c), and waist circumference (WC) were determinants of the cIMT, and only age, SBP, HBA1c, and blood urea nitrogen (BUN) were determinants of a cPS of > 2 in all individuals. Only HBA1c lost its association with regard to predicting the cIMT in non-diabetic subjects.

**Conclusions:**

HBA1c at > 5.9% can determine early and late stage subclinical atherosclerosis in community dwelling individuals, but only late stage subclinical atherosclerosis in non-diabetic subjects.

## Background

Carotid intima media thickness (cIMT) is a measure of the intima and media layers of the carotid artery. It is performed by B mode ultrasound in clinical practice. Hypertrophy of these intima or media (or both) layers results in a thicker cIMT. Factors that are responsible for this hypertrophy also develop or lead to progression of atherosclerosis [1]. The cIMT is considered to represent asymptomatic and subclinical atherosclerosis. This was studied and found to be a reliable and consistent marker of cardiovascular (CV) events in previous studies [1, 2]. It has been available to evaluate atherosclerosis and CV events since 2002 [3].

Studies also suggest that the carotid plaque score (cPS) alongside the cIMT is a valid marker of subclinical atherosclerosis and a strong predictor of CV disease (CVD) [4, 5]. Thickening of the cIMT reflects early stages of atherosclerosis, but plaque formation indicates later stages [6].

Glycosylated hemoglobin (HBA1c) was proposed as a screening and diagnostic marker for type 2 diabetes mellitus (T2DM) [7], as it can be used to determine the level of blood sugar over a long period of time and is also highly correlated with long-term complications of T2DM [8] and is associated with CVD in diabetic patients [9, 10]. It was found to be an important determinant of subclinical atherosclerosis, such as carotid atherosclerosis in T2DM [11]. Although it is widely used in diabetic patients, some epidemiological studies suggested an association between HBA1c and CVD in non-diabetic populations [12, 13], but other studies failed to reach this conclusion [14, 15]. Thus, there is uncertainty if HBA1c is correlated with subclinical atherosclerosis in non-diabetic patients or if it can be used to predict the cIMT.

To date, only a few studies have investigated relationships of the Uric acid level (URCA), HBA1c, and other tests with factors of subclinical atherosclerosis, such as the cIMT and cPS [16–19]. As there is controversy regarding these relationships in the general population and also in non-diabetic patients, the main objectives of this study were to elucidate these correlations and try to find a model to predict the cIMT and a high cPS based on patient characteristics and laboratory results and identify patients at high risk for developing CVD, both in community dwelling and non-diabetic individuals. Not all the community dwelling individual has the opportunity or necessity of performing a carotid duplex ultrasound to find out the status of subclinical atherosclerosis, so a model based on the characteristics and routine laboratory results may help to identify high risk patients for subclinical atherosclerosis to send out for further analysis.

## Methods

**Fig. 1:**
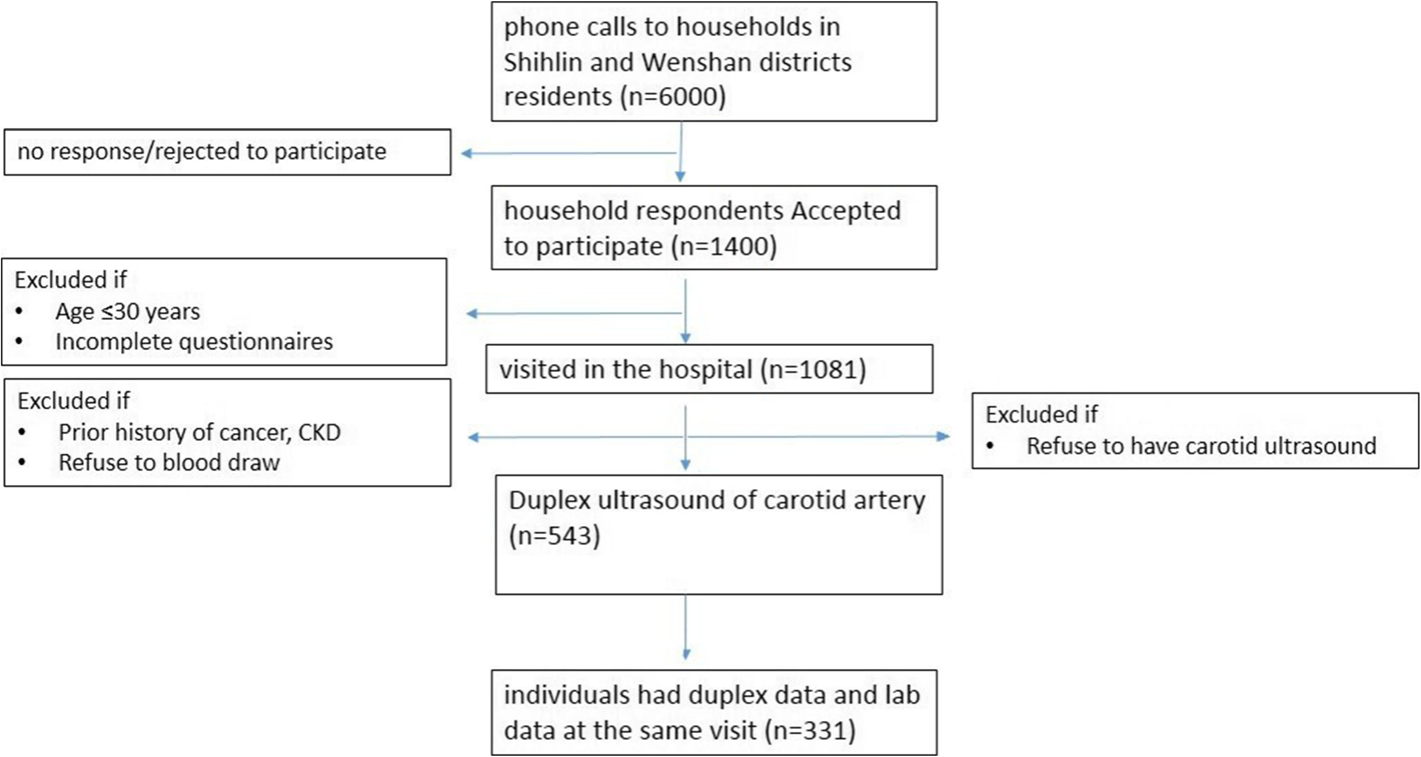
Flowchart for selection of the study participants. CKD: Chronic Kidney Disease

Data of individuals from a community-based prospective cohort study investigating CV and cerebrovascular risk factors on residents of 6 Villages in Shihlin District, and 6 villages in Wenshan District that are in the coverage of Shin Kong Wu Ho-Su Memorial Hospital and Wan Fang Hospital respectively in 2005 and 2006 were used in this study. Six thousand phone calls were made using the area codes of these districts. Respondents who consented to enter the study, asked about the demographic information and they were asked for the permission of keeping their contact information and to call upon them to come to the respective hospitals for further examination. Exclusion criteria were: age ≤ 30, incomplete questionnaire, prior history of cancer, chronic kidney disease, refusing to blood draw or duplex ultrasound. Individuals that had both duplex records and laboratory blood test results at the same visit were accepted in our study (Fig. 1). All study subjects signed a consent form in order to enter the original study, and no names were published in the results.

Simple and descriptive statistics (numbers and percentages for categorical variables and the mean and standard deviation (SD) for continuous variables) and analysis of categorical variables based on age group (< 45, 45~ 60, and > 60 years) and sex were carried out with Fisher’s exact test, and continuous variables were analyzed with an analysis of variance (ANOVA) and a post-hoc (least significant difference) test. Data including demographic characteristics, such as age, sex, age (years), height (cm), weight (kg), waist circumference (WC; cm), body-mass index (BMI; kg/m^2^), a history of stroke (STR), history of T2DM, and CVD, systolic (SBP) and diastolic blood pressure (DBP) (mmHg), pulse (beats/min), and also laboratory data including fasting blood sugar (FBS, mg/dl), uric acid level (URCA, mg/dl), blood urea nitrogen (BUN, mg/dl), creatinine (Cr, mg/dl), aspartate aminotransferase (AST, units/L), alanine transaminase (ALT, units/L), triglyceride (TRG, mg/dl), cholesterol (CHOL, mg/dl), low-density lipoprotein (LDL, mg/dl), high-density lipoprotein (HDL, mg/dl), C-reactive protein (CRP, mg/L), and glycated hemoglobin (HBA1c; %) were gathered using standard methods (using an X-1500-Sysmex, Deckman AU5800 and Tosho HLC-723G8 Automated glycol-hemoglobin analyzer).

Ultrasound of the carotid and vertebral arteries was done with a B-mode Duplex ultrasound (SONO 5500, HP, USA) and the following measurements were obtained for every individual: flow (ml), end diastolic velocity (EDV; cm/s), and peak systolic velocity (PSV; cm/s) measured in one cardiac cycle. The mean velocity (MV) and resistance index (RI) were calculated using these respective formulas: MV = (PSV + 2*EDV)/3 and RI = (PSV-MV)/PSV. The diameter of the cervical portion of the common carotid artery (CCA), the internal carotid artery (ICA) beyond the carotid bulb, and the external carotid artery (ECA) and vertebral artery (VA) sequentially on both sides and the number of plaques in the carotid arteries were measured by a cardiologist. The cPS in each person was calculated by adding the numbers of plaques on the right and left sides.

The cIMT was assessed at 1 cm from the carotid bulb on the left and right sides. The mean flow and RI were determined for every subject by taking the average of all flows and RIs of the left and right carotid and vertebral arteries. The mean cIMT and diameter of each artery were calculated by taking the average of the left and right sides in each individual.

Cutoff points for the cPS, cIMT, HBA1c, and SBP were set at the level of the 75% quartile of all subjects’ data. A simple Pearson’s correlation was calculated, and variables with significant correlations with the cIMT were included in a multiple linear regression to predict the cIMT. Two logistic regressions were done using variables with a significant correlation with the cIMT and cPS to calculate the odds ratio (OR) and 95% confidence interval (CI) for predicting a thick cIMT and a high cPS (using 75% percentiles as the cutoff points). Receiver operating characteristic (ROC) curves were drawn, and the Mann-Whitney test, ORs, and CIs were used to determine the significance. Multiple and logistic regressions of non-diabetic individuals were used to clarify the role of T2DM in determining the cIMT and high cPS. Observations were deleted due to missing values for the response or explanatory variables in all models. The alpha error was set to 0.05, and we used SAS version 9.4 (SAS, Cary, NC, USA) for all data analyses.

## Results

There were 150 (46.9%) women and 170 (53.1%) men with a mean age of 57.06 (range 32~ 85) years and a mean BMI of 23.55 kg/m^2^. The mean SBP and DBP were 123.57 and 78.02 mmHg, respectively. They had 0, 1, 2 (25%, 50%, and 75% quartiles, respectively) cPSs, with a minimum of 0 and a maximum of 17. The mean cIMT was 0.67 (range 0.43~ 1.125) cm. The 25%, 50%, and 75% quartiles were 0.59, 0.66, and 0.75 cm, respectively. Demographic, duplex, and laboratory data of subjects are presented in Table 1 categorized based on sex and age groups.

**Table 1:** Characteristics of community dwelling individuals, stratified by sex and age group

Variable	*N*	Mean ± SD Number (%)	Age group (years)				Sex		
			< 45	45~ 60	> 60	*p*	Male	Female	*p*
Total	–	–	40	150	129	< 0.0001	150 (46.9%)	170 (53.1%)	0.26
Age (years)	320	57.06 ± 10.85	38.7 ± 3.6^ab^	52.8 ± 3.9^c^	67.7 ± 5.5	< 0.0001	58.89 ± 11.73	55.43 ± 9.75	0.0044
Height (cm)	320	160.99 ± 8.49	165.4 ± 9.0 ^ab^	160.5 ± 7.8	160.2 ± 8.7	0.0019	167.20 ± 6.77	155.52 ± 5.61	< 0.0001
Weight (kg)	320	61.28 ± 10.78	64.9 ± 12.4^a^	59.7 ± 10.9	61.9 ± 9.8	0.0182	65.54 ± 9.60	55.75 ± 8.52	< 0.0001
WC (cm)	320	79.46 ± 9.74	78.9 ± 10.0^b^	76.9 ± 9.7^c^	82.6 ± 8.8	< 0.0001	85.00 ± 8.10	74.56 ± 8.35	< 0.0001
BMI (kg/cm^2^)	320	23.55 ± 3.10	23.6 ± 3.3	23.1 ± 3.3^c^	24.0 ± 2.7	0.0451	24.13 ± 2.88	23.03 ± 3.21	0.0014
FBS (mg/dl)	320	95.37 ± 21.86	91.7 ± 28.9	93.0 ± 19.2^c^	99.3 ± 21.9	0.0314	99.23 ± 24.33	91.96 ± 18.83	0.0029
URCA (mg/dl)	320	5.59 ± 1.44	5.6 ± 1.6	5.4 ± 1.4 ^c^	5.9 ± 1.4	0.0140	6.37 ± 1.30	4.91 ± 1.18	< 0.0001
BUN(mg/dl)	296	14.13 ± 4.05	11.4 ± 3.4 ^ab^	13.6 ± 3.4^c^	15.8 ± 4.4	< 0.0001	15.38 ± 4.06	13.23 ± 3.87	< 0.0001
Creatinine(mg/dl)	296	0.92 ± 0.63	0.8 ± 0.2^b^	0.8 ± 0.2^c^	1.1 ± 1	0.0046	1.13 ± 0.90	0.75 ± 0.14	< 0.0001
AST(units/L)	320	24.45 ± 15.38	20.3 ± 4.8	25.4 ± 20.7	24.7 ± 8.8	0.170	25.94 ± 20.96	23.14 ± 7.44	0.104
ALT(units/L)	320	23.99 ± 16.75	20.37.6	25.3 ± 19.9	23.7 ± 14.6	0.242	27.30 ± 21.78	21.07 ± 9.65	0.0008
TGL(mg/dl)	320	118.23 ± 76.60	125.2 ± 110.2	111.1 ± 64.3	124.5 ± 77.4	0.288	128.31 ± 87.56	109.34 ± 64.40	0.0269
CHOL (mg/dl)	320	203.66 ± 33.21	196.8 ± 37.6	204.8 ± 32.7	204.5 ± 32.4	0.372	200.25 ± 30.94	206.68 ± 34.90	0.0840
LDL(mg/dl)	320	135.81 ± 34.08	129.6 ± 36.8	135.6 ± 33.8	138.2 ± 33.5	0.371	136.08 ± 31.55	135.56 ± 36.26	0.891
HDL(mg/dl)	320	47.47 ± 13.63	45.7 ± 12.6	49.7 ± 13.6^c^	45.3 ± 13.6	0.0161	42.48 ± 12.73	51.88 ± 12.88	< 0.0001
CRP(mg/L)	320	0.188 ± 0.626	0.15 ± 0.2	0.13 ± 0.2	0.26 ± 0.9	0.256	0.196 ± 0.39	0.181 ± 0.77	0.823
HBA1c (%)	296	5.75 ± 0.959	5.7 ± 1.6	5.6 ± 0.6^c^	6 ± 1.0	0.0243	5.88 ± 1.11	5.69 ± 0.82	< 0.0001
Stroke	316	8 (2.5%)	0	2 (1.3%)	6 (4.8%)	0.109	5 (3.4%)	3 (1.8%)	0.50
HTN	320	105 (32.8)	2 (5%)	37 (24.7%)	66 (51.2%)	< 0.0001	61 (40.7)	44 (25.9%)	0.00
DM	320	38 (11.5%)	2 (5%)	12 (8%)	24 (18%)	0.0086	27 (18%)	11 (6.5%)	0.00
CVD	313	49 (14.8%)	3 (7.5%)	21 (14.6%)	25 (19.5%)	0.166	24 (16.2%)	25 (15.1%)	0.87
SBP(mmHg)	320	123.57 ± 18.95	112.2 ± 10.8^ab^	119.9 ± 17.8^c^	131.5 ± 19.2	< 0.0001	128.10 ± 16.15	119.58 ± 20.34	< 0.0001
DBP(mmHg)	320	78.02 ± 10.67	74.4 ± 8^ab^	78.2 ± 11.5	79.0 ± 10.2	0.057	80.25 ± 10.10	76.05 ± 10.80	0.0004
Pulse (beats/min)	320	72.21 ± 32.39	73.9 ± 8.5	73.2 ± 30.4	70.5 ± 38.9	0.747	74.78 ± 46.18	69.95 ± 9.48	0.183
Mean flow (ml)	329	219.80 ± 35.91	232.4 ± 28.3^b^	226.6 ± 37.2^c^	207.8 ± 33.9	< 0.0001	223.59 ± 35.89	216.33 ± 36.19	0.0742
Mean RI	322	0.656 ± 0.042	0.66 ± 0.037^a^	0.64 ± 0.042^c^	0.67 ± 0.045	< 0.0001	0.668 ± 0.042	0.646 ± 0.040	< 0.0001
Mean ECA DIA (cm)	331	0.357 ± 0.034	0.353 ± 0.029^b^	0.352 ± 0.037^c^	0.365 ± 0.033	0.0036	0.370 ± 0.033	0.346 ± 0.032	< 0.0001
Mean ICA DIA (cm)	331	0.429 ± 0.030	0.419 ± 0.0348^b^	0.424 ± 0.286^c^	0.439 ± 0.028	< 0.0001	0.443 ± 0.028	0.416 ± 0.026	< 0.0001
Mean CCA DIA (cm)	331	0.577 ± 0.056	0.554 ± 0.0382^b^	0.566 ± 0.0506^c^	0.599 ± 0.0614	< 0.0001	0.599 ± 0.055	0.558 ± 0.506	< 0.0001
Mean VA DIA (cm)	325	0.316 ± 0.030	0.313 ± 0.0323	0.314 ± 0.0306	0.321 ± 0.0296	0.104	0.324 ± 0.030	0.309 ± 0.0285	< 0.0001
cPS	331	1.86 ± 2.90	0.175 ± 0.594^b^	0.893 ± 1.663^c^	3.659 ± 3.596	< 0.0001	2.55 ± 3.45	1.35 ± 2.25	0.0002
Plaque presence	331	178(53.8%)	4(10%)	63(42%)	107(82.9%)	< 0.0001	95(63.3%)	79(46.5%)	0.003
cIMT (cm)	309	0.673 ± 0.110	0.571 ± 0.0762^ab^	0.647 ± 0.0849^c^	0.740 ± 0.1093	< 0.0001	0.698 ± 0.11	0.654 ± 0.103	0.0006
cIMT > 0.75	309	75(24.3%)	2(5.3%)	17(12.14%)	54(45%)	< 0.0001	42 (30.4%)	31 (19.2%)	0.03

*SD* standard deviation, *WC* waist circumference, *BMI* body-mass index, *DM* diabetes melitus, *HTN* hypertension, *CVD* cardio vascular disease, *SBP* systolic blood pressure, *DBP* diastolic blood pressure, *FBS* fast blood sugar, *URCA* uric acid level, *BUN* blood urea nitrogen, *AST* aspartate aminotransferase test, *ALT* alanine aminotransferase test, *TGL* triglyceride, *CHOL* cholesterol, *LDL* low density lipoprotein, *HDL* high density lipoprotein, *CRP* C-reactive protein, *HBA1c*, glycated hemoglobin, *cIMT* carotid intima media thickness, *RI* resistance index, *ECA* external carotid artery, *ICA* internal carotid artery, *CCA* common carotid artery, *VA* vertebral artery, *cPS* carotid plaque score

Results of Pearson correlations between the cIMT and different variables and also between the cPS and different variables are presented in Table 2. We entered each variable with a significant correlation with the cIMT in a linear regression model, and variables with high co-linearity were removed from the model using their variance inflation factor (VIF). Models were run for the logarithmic scale of the cIMT, as the cIMT was not normally distributed (Table 3).

**Table 2:** Pearson correlations of variables of interest with the carotid intima media thickness (cIMT) and carotid plaque score (cPS)

Variable	cIMT		cPS	
	*r*	*p*	*r*	*p*
Sex	0.196	0.001*	0.204	0.000*
Age	0.569	0.000*	0.538	0.000*
Height	0.18	0.759	0.052	0.356
Weight	0.168	0.004*	0.049	0.384
WC	0.333	0.000*	0.190	0.001*
BMI	0.229	0.000*	0.031	0.582
FBS	0.273	0.000*	0.120	0.031*
URCA	0.122	0.034*	0.134	0.017*
BUN	0.319	0.000*	0.323	0.000*
Cr	0.086	0.153	0.153	0.008*
AST	0.063	0.281	−0.019	0.740
ALT	0.091	0.117	−0.044	0.431
TGL	0.132	0.023*	0.072	0.197
CHOL	0.089	0.125	0.043	0.440
LDL	0.101	0.82	0.085	0.131
HDL	− 0.155	0.007*	−0.148	0.008*
CRP	0.084	0.145	0.022	0.698
HBA1c	0.332	0.000*	0.174	0.003*
Stroke	0.168	0.004*	0.114	0.043*
HT	0.423	0.000*	0.376	0.000*
DM	0.220	0.000*	0.159	0.004*
CVD	0.096	0.102	0.094	0.096
SBP	0.435	0.000*	0.316	0.000*
DBP	0.280	0.000*	0.069	0.216
Pulse	−0.006	0.917	−0.032	0.564
Mean flow	−0.146	0.011	−0.248	0.000*
Mean RI	0.167	0.004*	0.266	0.000*
Mean ECA DIA	0.215	0.000*	0.061	0.270
Mean ICA DIA	0.228	0.000*	0.200	0.000*
Mean CCA DIA	0.322	0.000*	0.330	0.000*
Mean VA DIA	0.016	0.785	0.084	0.129
Plaque score	0.542	0.000*	1	–
Plaque presence	0.485	0.000*	0.597	0.000*
cIMT	1	–	0.542	0.000*

*r*, Pearson correlation; *p*, *p* value; * *p* < 0.05

*WC* waist circumference, *BMI* body-mass index, *DM* diabetes melitus, *HTN* hypertension, *CVD* cardio vascular disease, *SBP* systolic blood pressure, *DBP* diastolic blood pressure, *FBS* fast blood sugar, *URCA* uric acid level, *BUN* blood urea nitrogen, *AST* aspartate aminotransferase test, *ALT* alanine aminotransferase test, *TGL* triglyceride, *CHOL* cholesterol, *LDL* low density lipoprotein, *HDL* high density lipoprotein, *CRP* C-reactive protein, *HBA1c* glycated hemoglobin, *cIMT* carotid intima media thickness, *RI* resistance index, *ECA* external carotid artery, *ICA* internal carotid artery, *CCA* common carotid artery, *VA* vertebral artery, *cPS* carotid plaque score

**Table 3:** Multiple linear regression for predicting Log (intima media thickness; cIMT)

Variable	Beta	Standarderror	*t* Value	Pr > |t|
Intercept	−1.34196	0.11238	−11.94	< 0.0001
Sex	0.01090	0.02033	0.54	0.5924
Age	0.00586	0.00080079	7.32	< 0.0001
WC	0.00228	0.00113	2.01	0.0452
URCA	−0.01027	0.00700	−1.47	0.1435
BUN	0.00326	0.00207	1.57	0.1166
HDL	− 0.00004201	0.00063481	−0.07	0.9473
HbA1c	0.03971	0.01228	3.23	0.0014
Stroke	0.02649	0.07207	0.37	0.7136
SBP	0.00165	0.00046219	3.58	0.0004
DM	−0.03006	0.03215	−0.93	0.3508

Linear model for predicting Log (cIMT), *F* value = 21.31, *p* < 0.0001, adjusted *r* squared = 0.413

*WC* waist circumference, *DM* diabetes melitus, *SBP* systolic blood pressure, *URCA* uric acid level, *BUN* blood urea nitrogen, *HDL* high density lipoprotein, *HBA1c* glycated hemoglobin

**Fig. 2:**
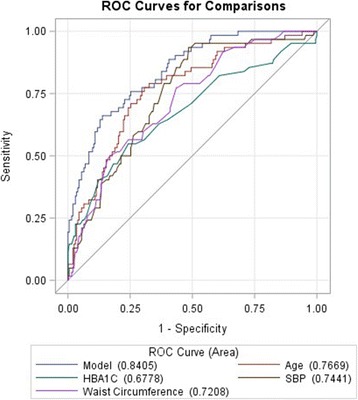
ROC curve for the logistic model for predicting a thick carotid intima media thickness (cIMT of > 0.75 cm) based on four independent variable of age, glycated hemoglobin (HBA1c), waist circumference, and systolic blood pressure (SBP)

**Fig. 3:**
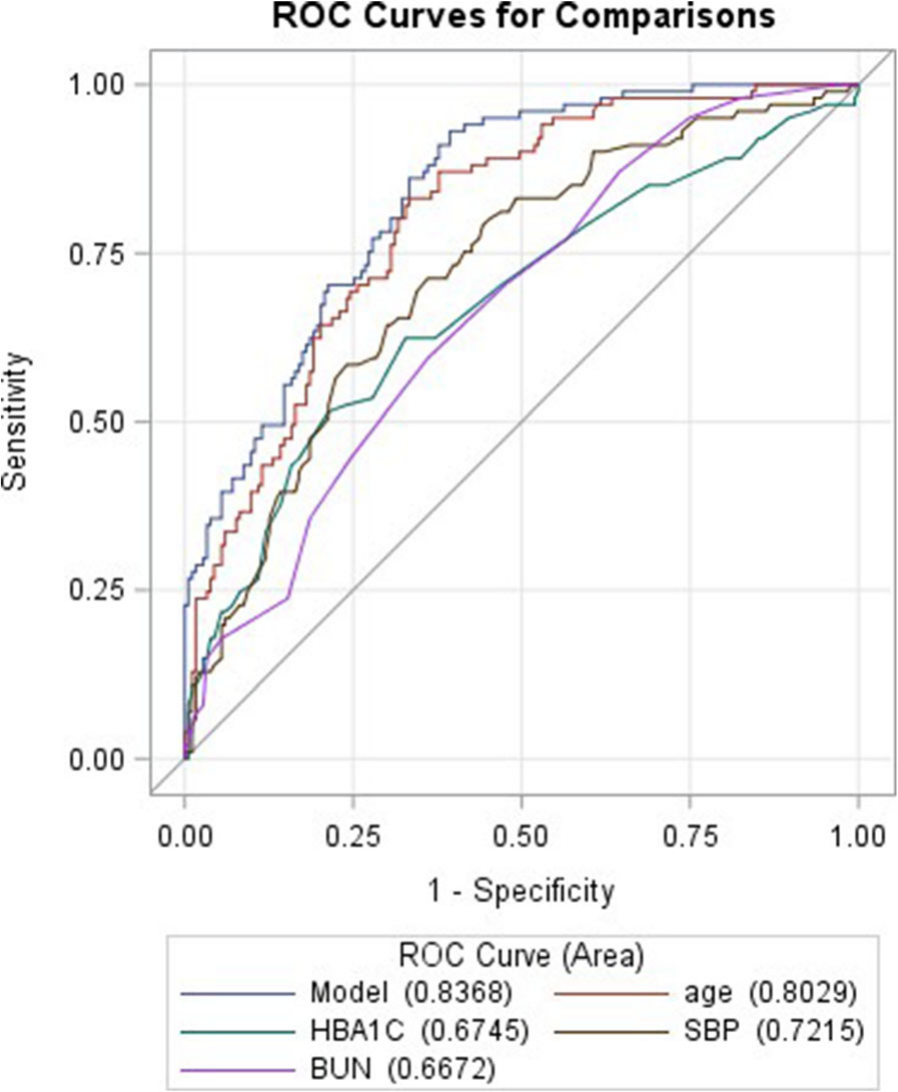
ROC curve for the logistic model for predicting a high plaque score (of > 2) based on four independent variable of age, glycated hemoglobin (HBA1C), systolic blood pressure (SBP), and blood urea nitrogen (BUN)

We used the third quartiles of WC (85.5 cm), HBA1c (5.9%), age (65 years), and SBP (135 mmHg) as cutoff points to run a logistic regression, and found the OR of the variables for predicting a thick cIMT (Table 4). We then ran a logistic regression model to predict a high cPS, and significantly correlated variables of age, sex, HDL, BUN, creatinine, HBA1c, URCA, T2DM and stroke history, SBP, and WC were included in the model; *p* values, ORs, and CIs of this model are also presented in Table 4. The ROC curves were drawn using the logistic regression to predict a thick cIMT and a high cPS in two models separately for each significant variable in previous logistic models (Figs. 2 and 3).

**Table 4:** Logistic regression model odds ratio for predicting a thick carotid intima media thickness (cIMT; of > 0.75 cm) and high plaque score (> 2) in all the individuals and in non-diabetic subjects

	cIMT> 0.75 in all the individuals			cPS > 2 in all the individuals			cPS > 2 in non-diabetic		
Variable	Odds ratio	95% Wald confidence interval		Odds ratio	95% Wald confidence interval		Odds ratio	95% Wald confidence interval	
Aged > 65 years	3.182	1.611	6.285	3.232	1.626	6.425	2.607	1.191	5.708
HBA1c > 5.9%	2.396	1.224	4.690	2.237	1.058	4.729	2.714	1.273	5.787
SBP > 135 mmHg	2.432	1.231	4.802	2.672	1.408	5.072	3.893	1.942	7.801
WC > 85 cm	3.458	1.766	6.770	0.972	0.932	1.013	–	–	–
Sex	–	–	–	0.919	0.389	2.169	1.235	0.499	3.055
Bun	–	–	–	1.103	1.013	1.201	1.115	1.014	1.226
Creatinine	–	–	–	3.846	0.564	26.234	1.965	0.259	14.903
HDL	–	–	–	0.982	0.958	1.006	0.986	0.962	1.010
Stroke	–	–	–	3.898	0.301	50.529	2.622	0.112	61.164
URCA	–	–	–	1.169	0.905	1.510	1.040	0.789	1.370
DM	–	–	–	1.406	0.494	3.998	–	–	–

*HBA1c* glycated hemoglobin, *SBP* systolic blood pressure, *WC* waist circumference, *BUN* blood urea nitrogen, *URCA* uric acid, *DM* diabetes mellitus

The ICA RI and CCA RI had significant correlations with the cIMT (*r* = 0.18748 and 0.22010, respectively, both *p* < 0.001), and the mean RI was correlated with the cIMT (Pearson *r* = 0.16738, *p* = 0.0036), but their relationship was not independent of age and sex, as this relationship disappeared after controlling for age and sex.

To further clarify associations of different variables in non-diabetic subjects, we ran second sets of Pearson correlations of HBA1c with cIMT and cPS and models to predict cIMT and cPS only in non-diabetic subjects. A significant correlation of HBA1c with the cIMT but not the cPS was present in T2DM subjects, and with both in non-diabetic subjects. After controlling for age, sex, SBP, and WC, however, HBA1c was not correlated with the cIMT in non-diabetic subjects (Table 5). However HBA1c could predict high cPS in non-diabetic individuals after controlling for age, SBP, sex, BUN, CR, HDL, stroke, and URCA (Table 4).

**Table 5:** Pearson correlation of glycated hemoglobin (HBA1c) with the carotid intima media thickness (cIMT) and plaque score (PS), and multiple regression for log (cIMT) in diabetic and non-diabetic individuals

	cIMT		cPS		Multivariate linear regression for log(cIMT)									
					WC		Sex		Age		SBP		HBA1c	
	*r*	*p*	*r*	*p*	b	*p*	b	*p*	b	*p*	b	*p*	b	*p*
Diabetic	0.35	0.049	−0.06	0.69	–	–	–	–	–	–	–	–	–	–
non-diabetic	0.196	0.002	0.158	0.012	0.0018	0.1	0.01	0.5	0.0059	< 0.0001	0.0005	0.0002	0.021	0.27

Adjusted *r*-squared for non-diabetic =0.365; *WC* waist circumference, *SBP* systolic blood pressure

## Discussion

Data of carotid duplex parameters and certain laboratory tests of 331 individuals from the general population were examined for relationships. Sex, age, weight, WC, BMI, FBS, URCA, BUN, TGL, HDL, HBA1C, a history of stroke, hypertension, and diabetes, SBP, DBP, the mean RI of CCA, ICA, ECA, VA, diameters of the ECA, ICA, and CCA, and the cPS were correlated with the cIMT. But among them, only age, HBA1C, WC, and SBP remained significantly associated with the cIMT after controlling for other correlated factors. Sex, age, WC, FBS, URCA, BUN, creatinine, HDL, HBA1C, a history of stroke, hypertension, and diabetes, SBP, the mean RI, and diameters of ICA and CCA were correlated with the cPS. But among them only age, HBA1C, and SBP remained significantly associated with the cPS after controlling for other factors. Results also showed that HBA1c cannot serve as an independent factor for predicting the cIMT in non-diabetic subjects but could predict a high cPS in non-diabetic subjects.

**Fig. 4:**
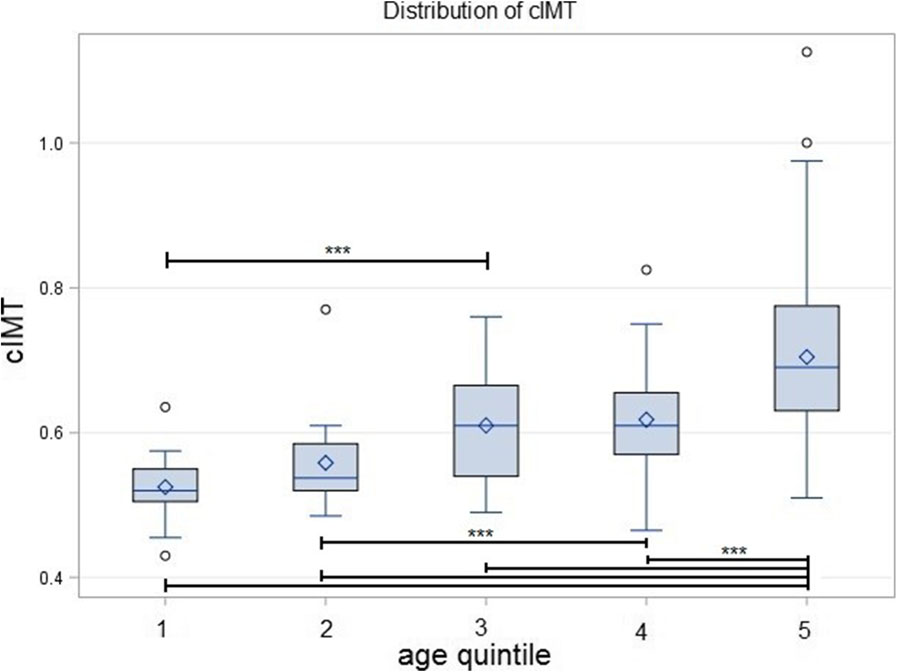
Box plots of the distribution of the carotid intima media thickness (cIMT) in different quintiles of age group. A significant difference is indicated by line and asterisks

We found a significant difference in cIMTs between men and women, and the cIMT was thicker in men. Our results are consistent with other studies [16, 17, 20]; however, sex does not independently predict the cIMT as confirmed by other studies [20]. On the other hand, we found a robust correlation between age and the cIMT, as it remained significant after controlling for other factors. Figure 4 shows that the mean cIMT was thicker in each age group compared to younger groups, and this difference was significant between some age groups. These findings are in accordance with other studies [16, 17, 20–22].

We found the BMI, TGL, HDL, FBS, and histories of CVD and T2DM to be factors correlated with the cIMT, and these results were consistent with those of Abbasi et al. [16], but we also found WC to be a stronger factor and independent determinant of the cIMT. Although some studies mentioned that LDL and TGL were correlated with the cIMT [20, 21], another study questioned this association, as there were no correlations of TGL and LDL with the cIMT [17]. We found no significant association of CRP with the cIMT or cPS. Although most studies confirmed this association [17, 21–25], Gao et al. [26] named it as an indirect determinant of the cIMT. We also found BUN to be an independent factor to identify individuals with a high cPS. Although Zhu et al. [27] did not find it to be a determining factor for thickening of the cIMT, it was correlated with the cIMT before running the multiple regression. Our results are consistent with theirs, except that we found a mild but significant association with a high cPS, even after adjusting for other variables.

URCA was found to determine subclinical atherosclerosis, as it independently predicted the cIMT in some studies [17], but other studies found no association [16, 28]. It was correlated with the cIMT in our study, but it failed to predict the cIMT or cPS after controlling for other variables.

**Fig. 5:**
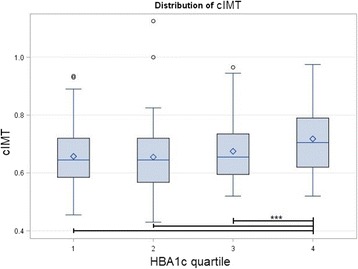
Box plots of the distribution of the carotid intima media thickness (cIMT) in different quartile of glycated hemoglobin (HBA1c). A significant difference is indicated by line and asterisks

Some studies found a correlation between HBA1c and the cIMT in diabetic patients [22, 24, 29]. Other studies [30, 31], on the other hand, found an independent association between these two factors, although Shah et al. [30] reported that this had a low prediction of variance (of < 20%). In studies of non-diabetic individuals [18, 19], no association was found, but Marini et al. [32] in a study of pre-diabetic patients versus non-diabetic controls proved otherwise. Some studies showed a strong association between the cIMT and HBA1c in people with normal glucose tolerance [33, 34]. And Huang et al. [33], in a study of a non-diabetic Chinese population, had similar results. Kowall et al. [35] found no association of glycemic measures such as FBS, HBA1c, and 2-h plasma glucose with the cIMT. Our study showed a significant correlation between the cIMT and HBA1c, and it remained significant even after controlling for many factors including the T2DM status. We also saw an increasing trend in cIMT levels in each HBA1c tertile, showing the strong association (Fig. 5).

Few studies have investigated the relationship between HBA1c and carotid plaque in non-diabetic individuals, and there are non-consistent results in the literature [33, 36]. Lee et al. [18] and Huang et al. [33] found a significant association between HBA1c and carotid plaque in non-diabetic people, but this relationship did not remain significant after adjusting for other factors. But Jorgensen et al. [35] found an independent relationship between these two measures. We also stratified subjects by the T2DM status and found correlations of the cIMT and cPS with HBA1c. We could not run a regression model on T2DM individuals due to the small sample size of T2DM subjects, but regression models on non-diabetic subjects showed that HBA1c could not independently predict the cIMT. However, this relationship was more robust regarding predicting a high cPS, as it was significant even after controlling for many variables (Tables 4 and 5). When we want to see the relationship between HBA1c and cIMT in non-diabetic subjects, our sample size decreases due to this stratification, so it is also probable that lack of association is because of the limited number of individuals in non-diabetic group. Our sample size in T2DM group is much smaller (*n* = 38) and it is probable that lack of correlation between HBA1c and high cPS is also due to this fact.

Our results suggest that HBA1c cannot predict the cIMT, but it can predict it with a high cPS in non-diabetic subjects. But as a general criterion, we can still use HBA1C to identify high-risk patients due to the ROC curves, and we can combine different variables to further increase the accuracy (Figs. 2 and 3).

Although it is rational to accept that carotid luminal enlargement can be used as a marker of atherosclerosis, as it should increase to preserve the space after hardening of the lumen and plaque formation, there is no proof whether these changes occur before the onset of diabetes [18], and more studies are needed to clearly discern this association.

Our study used no scoring system to compare the individuals for detection of subclinical atherosclerosis. So studies that can use the proposed variables with significant determination of early or late stage subclinical atherosclerosis to generate a scoring model would be recommended to further quantify the level of association. Additionally, status of the diabetes diagnosis, whether it is newly diagnose or not, and also the duration of diabetes, can potentially have some effects on our results and should be considered as a limitation in our study.

## Conclusions

In conclusion, HBA1c can be used alongside age, SBP, WC and BUN to identify individuals at high risk of early or late stage subclinical atherosclerosis from the community. HBA1c should be removed from our prediction model if one is dealing with early stage subclinical atherosclerosis in non-diabetic subjects, although HBA1c is useful in predicting late stage subclinical atherosclerosis in non-diabetic subjects.

## Abbreviations

ANOVA: Analysis of variance
BMI: Body mass index
BUN: Blood urea nitrogen
CCA: Common carotid artery
CHOL: Cholesterol
cIMT: Carotid intima media thickness
cPS: Carotid plaque score
CV: Cardiovascular
CVD: Cardio vascular disease
DBP: Diastolic blood pressure
ECA: External carotid artery
FBS: Fasting blood sugar
HBA1c: Glycated hemoglobin
HDL: High-density lipoprotein
HTN: Hypertension
ICA: Internal carotid artery
LDL: Low-density lipoprotein
Log: Logarithm with base 10
RI: Resistance index
SBP: Systolic blood pressure
SD: Standard deviation
T2DM: Type 2 diabetes mellitus
URCA: Uric acid level
VA: Vertebral artery
WC: Waist circumference
